# Acknowledgement to Reviewers of *Nanomaterials* in 2019

**DOI:** 10.3390/nano10010171

**Published:** 2020-01-19

**Authors:** Nanomaterials Editorial Office

**Affiliations:** MDPI, St. Alban-Anlage 66, 4052 Basel, Switzerland

The editorial team greatly appreciates the reviewers who have dedicated their considerable time and expertise to the journal’s rigorous editorial process over the past 12 months, regardless of whether the papers are finally published or not. In 2019, a total of 1807 papers were published in the journal, with a median time to first decision of 14 days and a median time from submission to publication of 33 days. The editors would like to express their sincere gratitude to the following reviewers for their generous contribution in 2019:
Abatzoglou, NicolasLehnherr, DanAbbrent, SabinaLeineweber, AndreasAbdala, AhmedLemordant, DanielAbdala, Paula MacarenaLemus, RanulfoAbdal-hay, AbdallaLenaghan, ScottAbdelkader, Amor M.Leon, FranciscoAbdel-Mohsen, A.M.Leonardi, FrancescaAbdulhalim, IbrahimLeonat, LuciaAbramovich, HaimLeong, David T.Abramovich, HaimLeonidas, PalilisAbramson, SébastienLeonyuk, N.I.Abu-Reziq, RaedLeporatti, StefanoAchilias, Dimitris S.Lepore, MariaAchim, CristinaLEPRINCE-WANG, YaminAcker, JörgLerouge, FredericAcres, Robert G.Lesch, AndreasAdachi, SadaoLessard, BenoitAdam, VojtechLeszczyńska, AgnieszkaAdamson, Douglas H.Létoublon, AntoineAdlhart, ChristianLettieri, MariateresaAdonin, Sergey A.Levchenko, IgorAflori, MagdalenaLevesque, Pierre L.Afzal, AdeelLevine, Mindy S.Agarwal, GunjanLewandowski, WiktorAgarwala, ShwetaLewkowski, JaroslawAgnoli, StefanoLeżańska, MariaAgrawal, PrashansaLgaz, HassaneAgrawal, RichaLhuillier, EmmanuelÁgueda, Vicente I.Li, GangAgustin, DominiqueLi, JianxiongAhadian, SamadLi, JiashenAhmad, ZeeshanLi, MeichunAhmadivand, ArashLi, Oi LunAhmadivand, ArashLi, Po-HsienAhmed, MaryaLi, WenyueAhmed, Mohammad BoshirLiakos, IoannisAhn, Byung TaeLiang, Chi-TeAhn, SanghyunLiang, YucangÀhniyaz, AnwarLianos, PanagiotisAkagi, SatoshiLiao, Ying-ChihAkazawa, HouseiLiberato, FerraraAkbari Garakani, MohammadLi-Destri, GiovanniÅkermark, BjörnLightfoot, PhilipAkhlaghi, ShahinLikodimos, VlassiosAkhtari, MassoudLim, Dong ChanAkita, SadanoriLim, Eun-KyungAkitsu, TakashiroLim, JiseokAkitsu, TakashiroLim, Min-CheolAla, GuidoLim, Yee YanAlarco, JoseLima, Sofia CostaAlavi, SamanLimongi, TaniaAl-Bataineh, SameerLin, Chia-FengAlberti, Marcos G.Lin, Chu-HsuanAlcoutlabi, MatazLin, Der-YuhAldakov, DmitryLin, LinhanAlegre, CinthiaLin, Yang-weiAlegria, Elisabete C.B.A.Lin, Yi-LiAlemán, CarlosLin, Yow-JonAlemany, PereLin, YuyuanAlessandri, IvanoLinares, N.Alexiev, UlrikeLinko, VeikkoAlexiou, ChristophLiopo, AntonAlgharagholy, LaithLisiecki, AleksanderAlhabeb, MohamedLisiecki, RadosławAlhwaige, AlmahdiLister, Tedd E.Ali Ansari, SajidLiu, XiangshengAli, MonsurLiu, YuAlikin, DenisLiu, YuxinAlmeida, JoseLiverani, LilianaAlmoazen, HassenLivi, SebastienAl-Shannaq, RefatLiVoti, RobertoAltmann, FriedrichLizundia, ErlantzAltomare, MarcoLlobet, EduardÁlvarez Centeno, TeresaLlorens, José ManuelAlvarez, JoséLo Presti, LeonardoÁlvarez, MarLöbmann, PeerAlvarez, Noe T.Locatelli, AndreaÁlvaro, CaballeroLohmus, RynnoAlzina Sureda, FrancescLolić, AleksandarAmarie, DragosLolla, DineshAmarpuri, GauravLong, Gary J.Amat, EsteveLonghi, MariangelaAmbrosetti, AlbertoLopes, Maria A.Ameloot, MarcelLópez Antón, RicardoAmendola, VincenzoLópez, Miguel ÁngelAnasori, BabakLópez-Cornejo, PilarAnastasescu, MihaiLopez-Jaramillo, Francisco JavierAndersen, Christian P.López-Lorente, Ángela InmaculadaAndersson, MatsLopez-Manchado, Miguel AngelAndo, YoshitoLópez-Rayo, SandraAndrade, LuísaLópez-Rubio, AmparoAndreoli, CristinaLopresti, FrancescoAndreou, ChrysafisLorite, Gabriela SimoneAndreou, ChrysafisLour, Wen-ShiungAndreozzi, AssuntaLourenço, JoãoAndres, JuanLow, NicholasAndrews, DavidLozano, R. M.Andritsch, ThomasLu, Ming-ChangAndrulevicius, MindaugasLub, JohanAng, Yee SinLubal, PřemyslAngelici, Robert J.Lubarda, VladoAngelova, AngelinaLuca, OanaAnicǎi, LianaLucena, RafaelAnifandis, GeorgeLuckham, PaulAniskevich, AndreyLuduena, RichardAnisoara Peptu, CatalinaLudwig, RalfAnnanouch, Fatima EzahraLuisetto, IgorAnni, MarcoLukáč, PavelAnnick, RubbensLukasik, RafalAntoine, RodolpheLukomska-Szymanska, MonikaAntoniac, IulianLukyanov, PavelAntoniadou, MariaLuna, CarlosAntonino, FamulariLundy, Fionnuala TAntonioli, DiegoLuty, TadeuszAntonopoulou, IoLützenkirchen, JohannesAntonović, AlanLützenkirchen-Hecht, DirkAoni, Rifat AhmmedLuzi, FrancescaApetrei, ConstantinLy, Thuc HueApetrei, ConstantinLyulin, Alexey V.Apollonio, FrancescaMa, TianyiApostol, MarianMaas, MichaelApostoluk, AleksandraMaasilta, IlariAquilano, DinoMacagnano, AntonellaAradilla, DavidMacak, JanAradilla, DavidMacaluso, RobertoArakawa, ToshiyaMaccaferri, NicolòArandiyan, HamidrezaMacdonald, ThomasAranguren, Mirta I.Machrafi, HatimArao, YoshihikoMacoas, ErmelindaArao, YoshihikoMacRaild, ChristopherAraujo, DanielMaeda, TomokiArbizzani, CatiaMaeng, Han-JooArena, AntonellaMagalhães, Fernão D.Argyle, MorrisMagasinski, AlexandreArias-Pardilla, JoaquínMagdanz, VeronikaAriese, FreekMaggioni, DanielaAriga, KatsuhikoMagnacca, GiulianaArmbruster, UdoMagnani, MauroArnaud, FrançoiseMagnucka-Blandzi, EwaArnold, Donna C.Magnusson, RobertArrais, AldoMahapatra, Manoj K.Arrieta, MarinaMahata, Manoj KumarArrigo, RossellaMahesh, SachithanadamArroyo, NetzahualcóyotlMahmoud, AbdelfattahArtetxe, BeñatMaisano, Jessica AAsadnia, MohsenMaiwa, HiroshiAsano, RyutaroMaiz, JonAsayama, ShoichiroMajak, JüriAsensio, Maria-CarmenMäkelä, Jyrki M.Ašmontas, SteponasMaki, YasuyukiAssadi, AymenMakkonen, LasseAssender, HazelMakovec, DarkoAsztemborska, MonikaMalandrino, Prof. GraziellaAta, SeisukeMalankowska, AnnaAtaollahi, NargesMalik, AdityaAtienzar, PedroMalik, RituAtsawasuwan, PhimonMalka, DrorAtuchin, Victor V.Malkin, AlexanderAvarvari, NarcisMallamace, DomenicoAversa, LucreziaMallavia, RicardoAvnir, DavidMalvić, TomislavAvolio, RobertoMalyi, Oleksandr I.Avram, MarioaraMamajanov, IrenaAwan, Shakil AhmedManakhov, AntonAxelevitch, AlexanderManavalan, BalachandranAyukawa, YasunoriMandelli, DavideAyyavoo, JayalakshmiManeiro, MarcelinoAznar, ElenaManera, Maria GraziaBabarao, RavichandarManfredi, LuigiBabeva, TsvetankaMani, Ganesh KumarBabu, AnishManian, Avinash P.Baccarani, GiorgioMankey, GaryBacsa, WolfgangMansoori, AliBadawy, Amro ElMantanis, GeorgeBadía Laíño, RosanaMarakatti, VijaykumarBadia, JoseMarasso, Simone LuigiBadilescu, SimonaMarcelli, Augusto ClaudioBae, ChangdeuckMarchal, Juan AntonioBaek, Jong-SuepMarchesan, SilviaBaek, YoungbinMarchiol, LucaBaeza, AlejandroMarconcini, PaoloBaeza, MireiaMarek, AntoninBagdziunas, GintautasMariani, StefanoBahr, DavidMarić, MilanBaimova, Julia A.Mariggiò, Maria A.Bajda, TomaszMarín, Josefa IsasiBaji, ZsófiaMarinkovic, DraganBakhtiyarov, SayavurMarinkovic, NebojsaBakolas, AsteriosMarino, AttilioBakonyi, ImreMárk, Géza IstvánBalandin, Alexander A.Markopoulos, AngelosBalandin, Alexander A.Markowska-Szczupak, AgataBalasingam, Suresh KannanMarkowski, Piotr MarekBalasubramanian, KannanMarler, JoanBaldi, FrancoMarmiroli, MartaBálint, ErikaMaroni, FabioBallarin, BarbaraMarotti De Sciarra, FrancescoBallato, JohnMarques, CarlosBallesta-Claver, JulioMarques, CarlosBallesté, Ruben MasMarques, Manuel Joaquim BastosBallesteros, BelénMarquez, Francisco MBalogh-Weiser, DianaMarradi, MarcoBalsamo, MarcoMarsal, Lluis F.Balzer, FrankMartel, BernardBanach, MarcinMartin, AidaBanchelli, MartinaMartin, Stephen M.Bandala, Erick R.Martines, EmilioBandyopadhyay, SupriyoMartinez Martinez, VirginiaBanfi, FrancescoMartinez Rodrigo, JavierBao, YiMartínez Rodrigo, JavierBaranowska, JolantaMartínez, CristinaBarawi, MariamMartínez, ElenaBarbato, Felicia Carla TizianaMartínez, Félix QuinteroBarbetta, AndreaMartinez-Calvo, MiguelBarbillon, GrégoryMartínez-Espinosa, Rosa MaríaBarbillon, GrégoryMartin-Garcia, BeatrizBarbu-Tudoran, LucianMartino, MarcoBardi, GiuseppeMartino, SabataBarek, JiriMartin-Ramos, PabloBarfidokht, AbbasMartins, José A.Barge, AlessandroMartins, RodrigoBarille, RegisMartins, Rui C.Barison, SimonaMartín-Sánchez, JavierBarker, Philip JMartín-Yerga, DanielBarnes, Frank S.Martone, AlfonsoBaron, MarcoMartucciello, NadiaBaroutaji, AhmadMarucci, AlvaroBarretta, RaffaeleMaruda, Radosław W.Barron, Andrew R.Masaharu, NakayamaBartley, JonathanMasayuki, SohgawaBartolomeo, Antonio DiMascini, MacelloBartsch, HeikeMassaro, MarinaBaruth, AndrewMastanjević, KristinaBaskoutas, SotiriosMastrangeli, MassimoBasoli, FrancescoMasuda, YoshitakeBatmunkh, MunkhbayarMasuo, SadahiroBatra, A. K.Matei, AndreeaBattiato, AlfioMaterer, Nicholas F.Batuk, MariaMathew, NithinBäumler, HansMathieu, DidierBayer, IlkerMatras-Postolek, KatarzynaBaykara, MehmetMatsuhisa, NaojiBayraktar, EminMatsui, IsaoBazylinska, UrzsulaMatsumoto, KozoBeake, BenMatsuno, TomonoriBear, Joseph C.Matsuo, TakashiBec, Krzysztof B.Matsushima, HisayoshiBecerra, MarleyMatsuura, TomoakiBechelany, MikhaelMatwijczuk, ArkadiuszBecucci, MaurizioMatykiewicz, DanutaBeg, MarijanMaupin-Furlow, Julie A.Behar, JoachimMauriello, FrancescoBeker, LeventMautner, AndreasBekyarova, ElenaMavrogonatou, EleniBelBruno, JosephMayer, DirkBeletskaya, Irina PetrovnaMayer, ThomasBelkhou, RachidMayr, PeterBella, FedericoMazur, MaciejBellardita, MariannaMba Blázquez, MiriamBellos, EvangelosMcCarty, Owen J.T.Bellucci, StefanoMcDonald, ThomasBelsley, Michael ScottMcGilly, Leo J.Beltsios, K.Medronho, Bruno Filipe FigueirasBender, FlorianMegiel, ElżbietaBenedetti, ElisabettaMehta, JodhbirBenes, HynekMei, BastianBenito, Juan M.Meijide, FranciscoBenoit, DenizotMeilhan, JéromeBenseman, Timothy M.Meixner, Alfred J.Benson, HeatherMekonnen, TizazuBerenjian, AydinMelchionna, MicheleBerlier, GloriaMele, ElisaBermudez, MariaMelin, FredericBernard, GeffroyMélinon, PatriceBernard, HUMBERTMeloni, EugenioBernardi, SaraMemoli, GianlucaBernasconi, LeonardoMenaszek, ElżbietaBernechea, MaríaMéndez, BianchiBert, NikolayMenegazzo, FedericaBertocci, FrancescoMeneghetti, GiovanniBertoglio, FedericoMenendez, BeatrizBertolotti, FedericaMenendez, MiguelBertolotti, MarioMenger, Fredric MBerton, PaulaMenini, MariaBertoncello, PaoloMenon, JyothiBERUETE, MIGUELMeo, MicheleBessergenev, Valentin G.Meo, Paolo LoBetancourt, TaniaMerabia, SamyBettini, SimonaMercado, Candy CBettotti, PaoloMercone, SilvanaBeyerlein, KennethMerle, BenoitBhandari, KhagendraMeroni, DanielaBhattacharjya, DhrubajyotiMeskinis, SarunasBhattacharya, SriparnaMeškys, RolandasBianchi, MassimilianoMessina, FabrizioBianco, VincenzoMestre, Ana S.Bielas, RafałMestres, NarcísBielecki, StanisławMeulenberg, Robert W.Biernat, JanMeyyappan, MeyyaBigall, NadjaMeziani, MohammedBigiani, LorenzoMezzi, AlessioBikiaris, DimitriosMiah, Abdul HalimBilewicz, AleksanderMiccio, FrancescoBini, FabianoMichail, ChristosBini, MarcellaMichalik, JanBirch, BrianMichalkiewicz, BeataBirke, Ronald L.Michel, Carlos R.Bishop, GregMichna, AnetaBison, GeorgMiculescu, FlorinBiswas, KaushikMiecznikowski, KrzysztofBittkau, KarstenMiederer, MatthiasBjork, EmmaMiglietta, Maria LuciaBjorklund, Robert B.Mikhelson, KonstantinBjörkman, TorbjörnMilanesio, MarcoBlanco, AngelesMilano, FrancescoBlanco, MirenMilano, GianlucaBlasi, PaoloMilasius, RimvydasBlau, WernerMilčius, DariusBlayo, AnneMilionis, AthanasiosBlein, ThomasMiluski, PiotrBłoch, KatarzynaMinak, GiangiacomoBlock, StephanMinakshi, ManickamBloh, Jonathan Z.Minami, IchiroBloise, AndreaMinea, Alina AdrianaBludov, YuliyMineva, TzonkaBobacka, JohanMínguez, Rebeca De NaldaBochenkov, Vladimir E.Minin, Igor V.Bochkarev, M. N.Miranda-Castro, RebecaBoczkaj, GrzegorzMiseki, YugoBoddapati, LoukyaMishra, Rupesh K.Bodzenta, JerzyMishra, YogendraBoeffel, ChristineMisyura, Sergey Y.Boffa, VittorioMitchell, AndrewBoffito, MonicaMitchell, JohnBoisseau, SebastienMitchenko, SergeBoix, Pablo P.Mitomo, HideyukiBokias, GeorgiosMitran, SorinBoldrini, StefanoMitropoulos, Athanasios C.Boles, CoreyMittova, Irina YaBolivar, Juan M.Miyazaki, KojiBoncel, SlawomirMizoshita, NorihiroBonduelle, ColinMizsei, JánosBonferoni, Maria CristinaMo, KunBonifácio, VascoMobbili, GiovannaBonnet, Célia S.Modica, MariaBonnet, Pierre-AntoineMoerman, DavidBonyár, AttilaMoghimi, SMBorensztein, YvesMohanta, AntaryamiBorfecchia, ElisaMohd-Yasin, FaisalBorges, João PauloMohles, VolkerBorges, JoelMoineau-Chane-Ching, Kathleen I.Borin, DmitryMoita, Ana SofiaBorowiec, JoannaMolina, C.B.Borriello, AnnaMoliner Martinez, YolandaBortolotto, TissianaMolla, Md. Ashraful Islam MollaBoscá, FranciscoMolle, AlessandroBoshir Ahmed, MohammadMoloney, Mark G.Bossmann, StefanMomeni, KasraBoudaden, JamilaMomida, HiroyoshiBouquillon, SandrineMonai, MatteoBouranis, Dimitris L.Mondal, SudipBourgeois, OlivierMonfort, OlivierBourillot, EricMonteiro Baptista, António PauloBourlinos, Athanasios B.Montgomery, Jason M.Bouropoulos, NikolaosMonti, AlessioBousser, ÉtienneMonticelli, OriettaBouyer, FredericMonzón, AntonioBouziotis, PenelopeMooers, BlaineBowen, JamesMoosmann, JulianBoyd, AnthonyMorais, SimoneBozo, EvaMorales, JuliánBraga, Susana SantosMoralli, DanielaBraic, MarianaMorant, CarmenBranicio, PauloMorasch, AlexanderBranquinho, RitaMorasso, CarloBreslin, Carmel B.Morávková, ZuzanaBrestic, MarianMorazzoni, FrancaBreunig, Hans GeorgMorbidelli, MassimoBrewer, Samuel M.Moreau, JulienBriesen, HeikoMoreno, FernandoBriois, PascalMoreno, InésBrogioli, DorianoMoreno, MiguelBrookes, NicholasMoretti, ElisaBrosseau, ChristianMorganti, PierfrancescoBrougham, DermotMori, WasukeBrousse, ThierryMorimoto, NobuyukiBrouzgou, AngelikiMorita, MasamuneBrown, David (France)Morkoc, HadisBrown, David (UK)Morley, Nicola ABrown, KatherineMoroni, GiovanniBroza, YoavMorozan, AdinaBrun, NicolasMorris, Jeffrey F.Bruno, AnnalisaMortazavi, BohayraBruno, MattosMoscone, DanilaBrutti, SergioMosk, AllardBryjak, MarekMotobayashi, KentaBubach, BaileyMott, Derrick M.Bubnov, AlexeyMourdikoudis, StefanosBuckner, Steven W.Mouzakis, Dionysios E.Bueken, BartMozia, SylwiaBueno-Alejo, CarlosMrázek, JanBufo, Sabino AurelioMrlík, MiroslavBugla-Płoskońska, GabrielaMrówczyński, RadosławBuha, JokaMróz, WaldemarBuhot, ArnaudMróz, ZenonBui, Thanh-TuânMrukiewicz, MateuszBulgariu, LauraMucalo, Michael R.Bunzli, Jean-ClaudeMucsi, ZoltánBuonocore, FrancescoMuench, FalkBuonomenna, Maria GiovannaMuenzenberg, MarkusBürgi, ThomasMuguruma, HitoshiBurguete, JavierMukherjee, SoumyaBurnat, BarbaraMukherjee, SudipBurns, Jorge S.Müller, BertBury, WojciechMüller, DannyBusby, YanMuller, GerhardBuschmann, Matthias H.Müller, GerhardBusquets, Maria AntòniaMüller, KarstenBussolati, OvidioMulmudi, Hemant KumarButler, JohnMuniz-Miranda, FrancescoByrne, HughMuniz-Miranda, MaurizioBystrov, VladimirMuñoz, MacarenaByun, JeehyeMuñoz-Batista, Mario JesúsCabaj, JoannaMunteanu, Florentina-DanielaCabaleiro, DavidMurase, NorioCabot, AndreuMuravyev, Nikita V.Cabot, Pere L.Murcia-López, SebastiánCabrales, LuisMusić, SvetozarCabrele, ChiaraMusmarra, DinoCacciotti, IlariaMuszyński, TomaszCadar, OanaMuziol, Tadeusz MikolajCadatal-Raduban, MarilouMyśliwiec, JarosławCadiz, FabianNa, KyungsuCahay, Marc M.Nabae, YutaCalabrese, LuigiNadolny, ZbigniewCalabrò, FrancescoNagai, MasayaCaldas, PauloNagai, NoriakiCalifano, ValeriaNagao, DaisukeCalvillo, LauraNagatani, NaokiCalvo, FlorentNagy, Dénes LajosCambedouzou, JulienNagy, EndreCamélia, Matei GhimbeuNair, AshwinCamerlingo, CarloNair, Devatha P.Cameron, DavidNair, Sandeep SudhakaranCameron, JosephNaito, KatsuyukiCametti, CesareNajlah, MohammadCamilli, LucaNakagawa, YoshinaoCaminati, GabriellaNakahara, YoshioCammas-Marion, SandrineNakamura, DaisukeCampbell, KrisNakashima, SeijiCampos-Martínez, J.Nalbach, PeterCandiani, GabrieleNamieśnik, JacekCaneschi, AndreaNanaki, StavroulaCanet Ferrer, JosepNanver, Lis K.Canet-Ferrer, JosepNarciso, JavierCanetta, ElisabettaNasiri, NoushinCanfield, BrianNataraj, LathaCangialosi, DanieleNattestad, AndrewCanle, MoisésNaumov, AntonCannas, MarcoNaumova, Ella A.Cannio, MariaNaushad, MCantalini, CarloNavalon, SergioCantele, GiovanniNavarro, RodrigoCao, XudongNayak, ArunimaCapasso, AndreaNeagu, AdrianCapelli, SofiaNeal, LukeCapeness, MichaelNeel, Ensanya A. AbouCapiglia, ClaudioNees, MatthiasCaporali, StefanoNeffe, Axel T.Capsoni, DorettaNegrete, GeorgeCapuano, RosamariaNegro, CarlosCaputo, GianvitoNeipp, CristianCaputo, GregoryNemes, Norbert M.Carabineiro, SóniaNemestothy, NandorCarata, ElisabettaNémeth, PéterCarbone, ClaudiaNemnes, George AlexandruCardia, Maria CristinaNeri, GiovanniCaridad, JoseNesterov, DmytroCarli, StefanoNesterova, Oksana V.Carlomagno, IlariaNeugebauer, DorotaCarmo, HelenaNevshupa, Roman ACarmona, FranciscoNgamchuea, KamonwadCarosio, FedericoNgene, PeterCarotenuto, GianfrancoNguyen, Duc ThanhCarrasco-Marin, FranciscoNguyen, Nhat TruongCartagena-Rivera, AlexanderNguyen-Tri, PhuongCaruntu, GabrielNi, GuangxinCasado-Coterillo, ClaraNi, MelodyCasanove, Marie-JoseNiaura, GediminasCasassa, SilviaNicholson, John W.Caseri, WalterNicoletta, Fiore PasqualeCasey, SeanNiemi, TapioCassani, Maria CristinaNiemirowicz-Laskowska, KatarzynaCassidy, John F.Nien, Yung-TangCastaldi, GiuseppeNienborg, BjörnCastaldo, AnnaNieszporek, KrzysztofCastanheira, Elisabete M.S.Nieto, AndyCastellanos-Gomez, AndresNiewa, RainerCastelli, Ivano E.Niidome, TakuroCastellino, MicaelaNikolelis, Dimitrios P.Castillo-Michel, HiramNikumb, SuwasCastro, EdisonNilges, TomCasula, MicheleNima, Zeid A.Cataldi, PietroNishikiori, HiromasaCatauro, MichelinaNishiyabu, RyuheiCauda, ValentinaNisnevitch, MarinaCavallaro, GiuseppeNistor, Ileana DenisaCavallini, MassimilianoNitschke, MirkoCavicchi, KevinNobili, AndreaCavigli, LuciaNocentini, SaraCazorla-Amorós, DiegoNofz, MarianneCazzanelli, MassimoNogueira, HelenaCecilia, Juan AntonioNogues, JosepCelano, UmbertoNomoto, TakahiroCelebrano, MicheleNomura, ShintaroCelzard, AlainNonomura, Dr. KazuteruCenteno, Miguel AngelNorek, MałgorzataCeresoli, DavideNorton, MichaelCerrato, Giuseppina PinucciaNoubactep, ChicgouaCesano, FedericoNouranian, SasanCesário, RuteNovikov, AndreiCeseracciu, LucaNovio, FernandoChagnes, AlexandreNovotna, VladimiraChai, Kyu YunNowak, SaschaChakraborty, SaumenNozomi, TakeuchiChampagne, BenoîtNtalii, NikolettaChandra, DhyanNtalli, Nikoletta G.Chandrasekaran, MurugesanNunes, Carla D.Chang, Chia-ChenNunes, DanielaChang, Dong WookNúñez Carmona, EstefaníaChang, Seung-CheolNurunnabi, MdChang, Sheng-PoNypelo, TiinaChantana, JakapanO’Mullane, AnthonyCharles, Andrew D. M.O’Rear, EdgarCharmas, BarbaraObata, YasukoChassaing, StefanObraztsov, Alexander N.Chaudhari, NitinO’Brien, Casey P.Chaudhary, Anna LisaOchi, MasayukiChaudhry, QasimOchiai, BungoChauhan, GarimaOchiai, TsuyoshiChava, Rama KrishnaOcłoń, PawełChavez-Angel, EmigdioO’Connor, BrianChen, BinlingOćwieja, MagdalenaChen, Chia-YunO’Duill, SeanChen, Hone-ZernOgasawara, HirohitoChen, Hui-YuOgasawara, ToruChen, I-Wen PeterOgawa, ShinpeiChen, Jem-KunOgino, IsaoChen, Jian-LianOh, JonghyunChen, JihuaOhara, KeishiChen, JinjuOhashi, MasashiChen, Kuan-RenOhlckers, PerChen, Lung-ChienOhsedo, YutakaChen, Pang-ShiuOkada, NarihitoChen, Szu-yuanOkamoto, HidekiChen, Tao-HsingOkamoto, HiroyukiChen, Wei-YuOkamoto, MasamiChen, YijiaOkazaki, JojiChen, Yu-ChihOkazaki, ToshiyaCheng, Fong-YuOkhrimchuk, AndreyCheng, Hsu-HuiOkochi, MinaCheng, HuanyuOlchowka, JacobCheng, YaOliveira, Mariana BragaCheraghian, GoshtaspOliveira, Paula A.Cherkaoui, KarimOliveira, VítorChiang, Anthony S.T.Ollila, SamuliChiang, Long YOlmos, DaniaChiavaioli, FrancescoOlsen, Lars FolkeChiolerio, AlessandroOlthuis, WouterChiono, ValeriaOmastová, MáriaChiu, Fang-ChyouOno, AtsushiChiu, Te-WeiOpitz, JörgChizari, KambizOrbea, AmaiaChizhik, Alexey I.Oreshonkov, AleksandrChmielarz, PawełOrge, CarlaChmielewski, AndrzejOrive, Alejandro GonzálezCho, Chang-HeeOrman, MehmetCho, Dae-HyunOrmeci, AlimCho, HannaOrobtchouk, RegisCho, In SunOrtega, José MarcosCho, Jung SangOrtenzi, Marco A.Cho, Kyu TaekOrtiz, AlfredoCho, Man HoOrtolani, LucaCho, So-HyeOsaki, TomohiroCho, SunghunOschatz, MartinChoi, Chul-JinOsipov, AndreyChoi, DukhyunOsipov, VladimirChoi, Hyo-JickOsses, NelsonChoi, HyoungOstasevicius, VytautasChoi, Jae-HongOstrauskaite, JolitaChoi, Jane RuOsvath, ZoltanChoi, Jeong-WooOsvet, AndresChoi, Si-YoungOta, SatoshiChoi, Yoon-SeukOtero, MartaChong, Parskson Lee GauOu, JianzhenChorazy, SzymonOuerghi, AbdelkarimChou, Jung ChuanOuyang, ZiChow, JamesOya, YuichiChowdhury, Ezharul HoqueOyama, KotaroChrissopoulou, KiriakiPacheco Tanaka, David AlfredoChristensen, Jørn BPaciorek-Sadowska, JoannaChronakis, Ioannis S.Packirisamy, MuthukumaranChroneos, AlexanderPacurari, MaricicaChrysikopoulos, ConstantinosPaczesny, JanChrzanowski, WojciechPadalkar, SonalChu, Ching-LiangPadamati, Ramesh BabuChul Wee, LeePadil, Vinod V. T.Chun, SechulPadnya, PavelChung, Chan-MoonPagano, StefanoChung, Dong YoungPagès, StéphaneChung, Ren-JeiPagliara, StefaniaChung, Sheng-HengPaik, TaejongCiampi, SimonePal, SudiptoCicala, GianlucaPalamà, IlariaCicciù, MarcoPalchoudhury, SoubantikaCinteza, Otilia LudmilaPalermo, EdmundCiocan, CorinaPalinko, IstvanCirillo, GiuseppePałka, NorbertCizek, JanPalkowski, HeinzClarke, Ronald JamesPallandre, AntoineClemens, OliverPallaoro, AlessiaCobianu, CornelPalma, LucaCoduri, MauroPalma, PauloCoelho, LuisPalmieri, ValentinaCoelhoso, IsabelPalomares, Antonio EduardoColace, LorenzoPalomba, ValeriaColangelo, GianpieroPalumbo, FabioCollins, ScottPalzer, StefanComan, CristinaPamies, RamonComfort, K KPana, OvidiuCompañ Moreno, VicentePanagopoulos, C. N.Comparelli, RobertoPandey, RaviConcepción, PatriciaPané Vidal, SalvadorConcetta Valeria Lucia, GiosafattoPanek, PiotrConcilio, SimonaPanek, RafalConnolly, James P.Panikkanvalappil, Sajanlal R.Constantin, LucianPanomsuwan, GasiditConte, ClaudiaPanzarasa, GuidoCordani, MarcoPaolicelli, GuidoCordoyiannis, GeorgePap, JózsefCorezzi, SilviaPapadimitriou, VassilikiCornelius, Thomas WalterPapadopoulos, Antonios N.Correa, Cinthia AntunesPapageorgiou, George Z.Corrigan, Damion KPapanikolaou, NikolaosCortés, Araceli GonzálezPapavasiliou, JoanCortese, BarbaraPapavassiliou, DimitriosCosco, DonatoPapini, EmanueleCosma, PinalysaPapoulis, DimitrisCosta, Carlos MiguelPappas, George S.Costa, PedroParajuli, DurgaCostantino, FerdinandoParakhonskiy, BogdanCostantino, LucaParaskevopoulou, PatrinaCostello, Ben De LacyPardanaud, CédricCoudert, Francois-XavierParigger, ChristianCousin, RenaudParisi, FilippoCoutinho, PaulaParisini, EmilioCoutinho, Paulo José GomesPark, Byung-WookCoy, EmersonPark, Chul-hoCozzoli, P. DavidePark, DaehwanCozzolino, Anthony F.Park, Deog-SuCragg, PeterPark, Deok-YongCredico, Barbara DiPark, Dong HyukCreton, StuartPark, EnochCrisp, Ryan W.Park, JaehoonCristallini, CaterinaPark, Jin GyuCristea, CeciliaPark, Jin KuenCristea, Mihaela DanaPark, Joo-CheolCristian, PetcuPark, Jung-hyunCritchley, KevinPark, JunyongCroce, Anna CletaPark, Kwi-IlCroissant, Jonas G.Park, Kwi-IlCrossley, AlisonPark, KyeongsoonCrucho, Carina Isabel CorreiaPark, MiraCrucianelli, MarcelloPark, Myung HwanCruz, SandraPark, SaeJuneCruz-Yusta, ManuelPark, SangmoonCsík, AttilaPark, SunggookCsoka, LeventePark, SunghaCsontos, MiklósPark, WansuCtibor, PavelPark, Young WookCuberes, TeresaParkinson, DilworthCucchiarini, MagaliParlea, LorenaCulshaw, BrianParlett, ChristopherCzeppe, TomaszParlińska-Wojtan, MagdalenaCzerwinski, AndrzejParmentier, JulienCzigany, ZsoltParnell, Andrew J.Czyż, JarosławParnian, Mohammad JavadD’Anna, FrancescaParsons, J.G.Da Costa Ribeiro, Ana PaulaParvez, KhaledDa Costa, Ana RosaPascual, LauraDa Silva, Jaime Pedro OliveiraPascuta, PetruDabrowiak, JamesPasini, DarioD’accolti, LuciaPasinszki, TiborDaeneke, TorbenPasquinelli, GianandreaDagdeviren, CananPassalacqua, RosalbaDahal, Rajendra PPastore, RobertoDal Col, JessicaPastrana-Martínez, Luisa M.D’Alfonso, LauraPatanè, SalvatoreDalmoro, AnnalisaPatelli, AlessandroD’Amora, UgoPathakoti, KavithaDa’na, EnshirahPatil, Santosh SDaniel-da-Silva, AnaPauliac-Vaujour, EmmanuelleDaniele, MichaelPauliukaite, RasaDanko, MartinPaulsen, HaukeDAnte, SilviaPavesi, MauraDanti, SerenaPavlatou, Evangelia A.Darling, Seth B.Pavlos, KlepetsanisDarmanin, ConniePawar, RadheshyamDarvell, BrianPawar, ShitalDas, NarottamPawlak, MichałDas, SanjibPawłat, JoannaDash, BirajaPawlus, PawełDaubon, ThomasPeale, RobertDavid, Iulia GabrielaPedraza, Luís CerdánDavim, J. PauloPedro Acuña, GuillermoDavis, FrankPegoretti, AlessandroDavis, FredPeijnenburg, Willie J. G. M.Davis, GrahamPelin, MarcoDawes, JudithPeljo, PekkaDawn, ArnabPelka, RafałDawson, James A.Pellegrino, FrancescoDawson, JonathanPelli, StefanoDe Aguiar, José BarrosoPeña, Jose IgnacioDe Andrés, AliciaPeng, RuoguDe Aza, Piedad N.Peng, ZhiliDe Bonis, AngelaPenkov, Oleksiy V.De Chaisemartin, LucPensabene, VirginiaDe Falco, GianluigiPeppas, GeorgiosDe Gisi, SabinoPeppel, TimDe La Escosura-Muñiz, AlfredoPerche, FedericoDe La Fuente, GermánPereira Gonçalves, AntónioDe La Rica, RobertoPereira, Cláudia C. L.De Lacalle, Luis N. LópezPereira, EulaliaDe Luca, LuigiPereira, LuísDe Luca, PierantonioPereira, Luiz Fernando RibeiroDe Marcellis, AndreaPeres, José A.De Nijs, BartPérez Comuñas, María JoséDe Oliveira, Mario AndersonPerez Del Pino, AngelDe Sio, LucianoPérez Menéndez, Ramón JoséDe Sousa, Célia TavaresPérez-Álvarez, LeyreDe Stefano, LucaPérez-Juste, IgnacioDe Vietro, NicolettaPérez-Mendoza, Manuel J.De Zotti, MartaPérez-Prieto, JuliaDeckert, AlicePérigo, Élio AlbertoDecroly, AndréPerinelli, Diego RomanoDeimede, ValadoulaPeris, José-EstebanDejam, MortezaPerkins, Laura E. L.Del Gaudio, CostantinoPerlepes, Spyros P.Delfino, InesPerles, JosefinaDell’Aglio, MarcellaPerrotti, VittoriaDel-Rio Celestino, MercedesPerumal, SugunaDelville, Marie-HélènePeshek, TimothyDemel, JanPestov, AlexanderDenat, FranckPetala, NatasaDeng, Hai-QiangPetersen, ElijahDepciuch, JoannaPetersen, EricD’Errico, GerardinoPeterson, Dominic S.Derrien, ThibaultPetkov, NikolayDesmaris, VincentPetrella, AndreaDeuss, PPetrič, MarkoDevadas, Mary SajiniPetrila, IulianDevasurendra, AmilaPetrò, StefanoDEVEL, MichelPetrova, Krasimira T.Dhakshinamoorthy, AmarajothiPetrovic, ZeljkaDhand, VivekPetrovykh, Dmitri YDhanjai, DhanjaiPetukhov, Andrei V.Dharavath, SrinivasPeveler, WilliamDhaygude, H. DPhilipose, UshaDi Bartolomeo, AntonioPhillips, JonathanDi Bella, SantoPhillips, LaurieDi Corato, RiccardoPhoenix, S. LeighDi Girolamo, RoccoPiasecki, AdamDi Marco, AlessandroPica, MonicaDi Martino, PieraPiccirillo, ClaraDi Noto, VitoPichon, CélineDiamanti, Maria VittoriaPieczka, AdamDiaz Fernandez, YuriPielichowska, KingaDíaz, EsperanzaPielichowski, KrzysztofDiba, ManiPiergiovanni, LucianoDichiara, AnthonyPierini, FilippoDickert, Franz L.Pietrasik, JoannaDickey, MichaelPifferi, ValentinaDíez, NoelPikulin, AlexanderDíez-Pascual, Ana MaríaPilot, RobertoDikin, Dmitriy A.Pina, JoãoDillert, RalfPina, SandraDimakis, NicholasPineda Rodríguez, TeresaDimitratos, NikolaosPineda, AntonioDimitrievska, MirjanaPineda, EloiDimos, KonstantinosPineiro, Manuel M.Dina, Nicoleta ElenaPingitore, NicholasDinca, ValentinaPinho, LuísDinh, ToanPini, ValerioDinh, Van TuanPinkas, IddoDini, DaniloPinto Da Silva, LuísDini, LucianaPinto, NicolaDinischiotu, AncaPiozzi, AntonellaDinu, Maria ValentinaPiperno, AnnaDiring, StephanePiperopoulos, ElpidaDistaso, MonicaPiro, BenoitDixon, David A.Pirri, AngelaDJANASHVILI, KristinaPiscitelli, FilomenaDo Rego, Ana Maria P. L. R. BotelhoPispas, StergiosDobrovolskaia, MarinaPistone, AlessandroDobrzynski, PiotrPiszczek, PiotrDocoslis, ArisPita, MarcosDodds, ChrisPittalà, ValeriaDohnal, FadiPitto-Barry, AnaïsDolocan, AndreiPixley, SarahDomb, AviPizzoferrato, RobertoDombek, GrzegorzPlonska-Brzezinska, MartaDomenici, ValentinaPlotniece, AivaDomine, Marcelo E.Plyushch, ArtyomDominguez, Carmen M.Pochard, IsabelleDong, YajiePochat-Bohatier, C.Donnet, ChristophePodlipná, RadkaDorosz, DominikPohl, PawelDorozhkin, Sergey V.Poitras, DanielDorp, Dennis VanPokharel, PashupatiDos Santos-García, Antonio JuanPokhrel, MadhabDoustkhah, EsmailPolat, Emre OzanDressel, MartinPolavarapu, LakshminarayanaDrevet, RichardPolimeni, AntonellaDrewniak, SabinaPolimeno, AntoninoDrikakis, DimitrisPolitano, AntonioDrisko, GlennaPolito, LauraDrlica, KarlPolowczyk, IzabelaDuboz, Jean-YvesPolyakov, NikolayDuguet, EtiennePoma, AlessandroDulski, MateuszPoma, AnnaDumee, LudovicPomastowski, Paweł PiotrDumitran, Laurenţiu MariusPons, JosefinaDumitrica, TraianPons, ThomasDumont, PierrePonzoni, AndreaDumur, FrédéricPopa, LacramioaraDurach, MaximPopielarz, RomanDurante, ChristianPorat, Ze’evDurinck, JulienPorrati, FabrizioDurndell, LeePorwal, HarshitDušková-Smrčková, MiroslavaPostigo, Pablo AitorDuta, LiviuPostnikov, PavelDutot, MélodyPotapov, AndreiDutta, KingshukPoumirol, Jean-MarieDuval, Raphaël E.Pourceau, GwladysDüzgünes, NejatPourrahimi, Amir MasoudDvoyashkin, MuslimPourret, OlivierDyatkin, BorisPourrezaei, KambizDyck, Ondrej E.Povolotskiy, Alexey V.Dyzia, MaciejPoyato, RosalíaDzakpasu, MawuliPradanos, PedroDzantiev, B. B.Pradhan, Dhiren K.Dzida, MarzenaPradhan, Sangram K.Dziedzic, ArkadiuszPrado-Gotor, RafaelDziubek, KamilPrakash Kottapalli, Ajay GiriEason, DavidPrassl, RuthEbara, MitsuhiroPrat, MariaEconomopoulos, Solon P.Praus, PetrEconomou, DemetrePredoi, DanielaEdgar, James H.Prida, Victor M.Edlund, UlricaPrieto, CarlosEfimov, AlexanderPrieto, CristinaEfstathopoulos, Efstathios P.Printz, Adam D.Eftekhari, AliProchazka, MarekEftimie Totu, EugeniaProchowicz, DanielEgawa, YuyaProfire, LenutaEgberts, PhilipProkopidis, KonstantinosEgli, RamonProkopowicz, MagdalenaEhrmann, AndreaPrudenzano, FrancescoEinarsson, ErikPruna, Alina IulianaEklund, PerPrytz, ØysteinEkuni, DaisukePtok, AndrzejEleni, MakaronaPu, Ying-ChihEl-Gendy, Ahmed A.Pucci, AndreaEligiusz, PostekPuga, Alberto V.Ellahi, RehmatPuglia, DeboraEl-Moghazy, AhmedPuglisi, RosariaEl-Safty, SherifPuiggalí, JordiEmoto, MakotoPuli, Venkata SreenivasEndoh, TamakiPullano, SalvatoreEnesca, Ioan AlexandruPum, DietmarEngelhart, AaronPunzo, FrancescoEngelke, HannaPuppi, Dr. DarioEngqvist, HåkanPurcar, VioletaEnnas, GuidoPuszkiel, JuliánEnnen, IngaPutkonen, MattiEnrico, Gherlone FelicePutla, SudarsanamEnríquez Pérez, EstherPutz, Mihai V.Enyashin, AndreyPyatenko, AlexanderEpifani, MauroPyrgioti, EleftheriaErbe, ArturQiang, ZheErcole, FrancescaQuagliariello, VincenzoErdem, EmreQuang Trung, TranEriksen, René LyngeQuémerais, BernadetteErmini, Maria LauraQuesne, MatthewErmolina, IrinaQuijada-Garrido, IsabelErwan, PaineauQuintanilla, AsuncionEscorihuela, JorgeQuinto, Emiliano JoséEsen, CemalQuynh Hoa, LeEslami, HosseinRaabe, GabrieleEsparza, PedroRabczuk, TimonEspiña, BegoñaRaccichini, RinaldoEsposito Corcione, CarolaRack, Philip D.Esposito, ElisabettaRadadia, Adarsh D.Esposito, GennaroRadhakrishnan, SivaprakasamEsposito, SerenaRadtke, AleksandraEspro, ClaudiaRadziuk, DaryaEstellé, PatriceRafiee, MohammadEstelrich, JoanRaga, Sonia R.Esteves Da Silva, Joaquim C.G.Ragab, TarekEstrany, FrancescRaganati, FedericaEtacheri, VinodkumarRagona, LauraEtienne, MathieuRagusa, AndreaEttayapuram Ramaprasad, Azhagiya SingamRahimi, ParvanehEubank, Jarrod F.Rahman, Md. MahbuburEuchner, HolgerRahme, KamilEugenio, María E.Rai, AmriteshEvangelisti, ClaudioRai, PrakashEvans, ChristopherRainer, AlbertoEvlyukhin, Andrey B.Rajapandiyan, PanneerselvamEvtugyn, GennadyRajendran, Jagathesh ChandraFabbri, FilippoRamakumar, KinthadaFabiano, EduardoRamanavicius, ArunasFabio, MarmottiniRamapuram, JayFabisiak, KazimierzRamirez-Castellanos, JulioFabre, BrunoRamirez-Vick, JaimeFàbrega, LourdesRamos Cabrer, PedroFagadar-Cosma, EugeniaRamos, DanielFagan, JeffreyRanc, VáclavFaggio, GiulianaRanjit, SumanFaia, PedroRanjkesh, AmidFajín, José L. C.Rankin, Rees B.Fakharuddin, AzharRanoszek-Soliwoda, KatarzynaFakhrullin, Rawil F.Ranzato, EliaFalco, AlbertoRaposo, MariaFales, AndrewRasheed, TahirFaley, MichaelRashid, Mohammad MamunurFalko, VladimirRaspanti, MarioFancher, Chris M.Ratnakumar, B. V.Fanciulli, CarloRaty, Jean-Yves RatyFang, ChengRaucci, Maria GraziaFang, Ronnie H.Raudino, AntonioFarajpour, AliRault-Berthelot, JoëlleFare, SilviaRauwel, ErwanFarè, SilviaRauwel, ProtimaFarka, ZdeněkRavelli, DavideFarris, StefanoRavnsbæk, Dorthe B.Farrugia, BrookeRavula, SudhirFasano, MatteoRazdolski, Ilya E.Fateixa, Sara Isabel AugustoRead, JeffreyFaulkner, R. G.Reber, ChristianFavre-Réguillon, AlainRebollar, EstherFazzolari, FiorenzoReddy, M. VenkatashamyFeczkó, TivadarReeves-McLaren, NikFedorov, GeorgyRego, GasparFeis, AlessandroRegulska, ElzbietaFelgueiras, HelenaReimhult, ErikFelicetti, FedericaReinhard, FriedemannFélix, LuisReinosa, Julián JiménezFeliz, MartaReipa, VytasFelner, IsraelReis, Paulo Nobre Balbis DosFenyvesi, EvaRej, SouravFernandes, Maria HelenaReli, MartinFernandes, Mariana Sofia PeixotoRemes, Mgr. ZdenekFernández Ramos, María DoloresRene, Eldon R.Fernández, AdolfoRenman, GunnoFernández, José A.Rennhofer, HaraldFernández-García, MartaRenò, FilippoFernández-Romero, Juan ManuelRerat, MichelFerrara, ChiaraRestivo, JoãoFerrari, LucReukov, VladimirFerrari, MaurizioReverberi, Andrea P.Ferrari, MaurizioReyes-Ortega, FelisaFerrari, MicheleRhee, Jong IlFerraris, SaraRibeiro, ClarisseFerraro, PietroRibeiro, DanielaFerreira, HelenaRibis, JoelFerreira, Jose M.F.Riccardo, CarloFerreira, QuirinaRicci, MarilenaFerreiro, Anxo FernándezRiccò, RaffaeleFerrer, BelenRichard, CyrilleFerrero, Guillermo Á.Richard, MandleFerretti, Anna MariaRichardson, JosephFiandra, LuisaRichtera, LukášFic, KrzysztofRider, Andrew N.Ficek, MateuszRidi, FrancescaFierro, VanessaRiedl, BernardFilgueira, CarlyRiela, SerenaFilip, JaroslavRiess, GisbertFilipovic, NenadRiha, Shannon C.Finney, Eric E.Rim, Kyung TaekFioravanti, AmbraRim, You SeongFiorenza, PatrickRimondini, LiaFioretti, BernardRinkevich, AnatolyFiori, StefanoRioual, S.Fiorillo, AntoninoRivadeneyra, AlmudenaFiorillo, FaustoRiveiro, AntonioFischer, HolgerRizal, ConradFlandin, LionelRiziotis, ChristosFlater, Erin E.Rizzi, Gian AndreaFlieger, JolantaRizzo, AntonioFlorczyk, StephenRizzo, CarlaFlorea, IleanaRizzolio, FlavioFloris, FrancescoRoberts, NicholasFlox, CristinaRobinson, Donald A.Focsan, MonicaRocai Cabarrocas, PereFoelske, AnnetteRocchitta, GaiaFologea, DanielRodrigo, Sergio GFomin, Vladimir M.Rodríguez Bernaldo De Quirós, AnaFonseca, AnaRodriguez Diaz, Camilo ArturoFont, FrancescRodriguez, JavierFontana, AntonellaRodriguez, NoelFontana, FlaviaRodríguez, RafaelFontanesi, ClaudioRodríguez-Jerez, José JuanFonte, PedroRodríguez-López, JoaquínFormela, KrzysztofRodriguez-Lorenzo, LauraFornasiero, PaoloRogala, MaciejFornell, JordinaRoh, ChanghyunForner, LeopoldoRojo, LuisFortenberry, RyanRomanato, FilippoFortuna, AnaRoma-Rodrigues, CatarinaFortunati, IlariaRomeo, FrancescoFortunato, GiuseppinoRomero, Francisco J.Foster, Christopher W.Romero, IsabelFracasso, GiulioRomero-Enrique, Jose M.Fraga-García, PaulaRomero-Salguero, Francisco J.Fragoso, AlexRoppolo, IgnazioFraix, AuroreRosado, CatarinaFrancés-Soriano, LauraRossella, FrancescoFrancia, CarlottaRossetti, IleniaFranco, AlessandroRostovtsev, YuriFrank, IrmgardRouhani, ShamilehFrankcombe, TerryRowe, AlistairFranssila, SamiRoy, AnupamFratoddi, IlariaRoy, SagarFrattini, DomenicoRozga, PawelFrederick, Brian G.Rozynek, ZbigniewFreedman, KevinRtimi, SamiFreeman, EricRubino, Federico MariaFrey, MargaretRuda (FRSC), HarryFriák, MartinRudzinski, JosephFrielinghaus, HenrichRudziński, WojciechFrisenda, RiccardoRuffino, FrancescoFröhlich, EleonoreRuggieri, FabrizioFrontistis, ZachariasRuggiero, AlessandroFuierer, PaulRuiz De Larramendi, IdoiaFujii, TakenoriRuman, TomaszFUJIWARA, AkihikoRumpf, KlemensFukuda, TakeshiRumyantseva, MarinaFulvio, Pasquale FernandoRurali, RiccardoFurumi, SeiichiRussell, ThomasFurusawa, HiroyukiRusso, Patrícia A.Furuta, HiroshiRusso, PietroFuso, FrancescoRusso, SaverioGabilondo, NagoreRusso, TeresaGaczynska, MariaRuths, MarinaGadde, SureshRyabchikova, Elena I.Gadisa, Abay DinkuRyan, James G.Gadomski, AdamRybak, AndrzejGaffet, EricRybiński, PrzemysławGafurov, MaratRydosz, ArturGago, SandraRydz, JoannaGalamboš, MichalRyu, Ja-HyoungGaletti, MariclaRyu, JehwangGalian, Raquel E.S. Oh, DanielGalinetto, PietroSabirov, DenisGallei, MarkusSaccà, AdaGalstyan, VardanSacco, AdrianoGambardella, AlessandroSacco, OlgaGamelas, JoséSada, KazukiGammer, ChristophSadowska, BeataGamsjäger, ErnstSadreev, Almas F.Gane, PatrickSafa, KasapGanesan, PalanivelSafaei, Mohammad RezaGangishetty, MaheshSafdari Shadloo, MostafaGanguly, PritamSafiuddin, Md.Ganikhanov, FeruzSagué, Anna LaromaineGanta, DeepakSaha, GobindaGantenbein, BenjaminSahoo, SumanthaGao, NanSahu, Saura C.Gao, XuefeiSaielli, GiacomoGao, YongfengSaijo, YoshifumiGarbovskiy, YuriySaini, NaurangGarcía Rodríguez, JuanSaito, GenkiGarcía Ruiz, Francisco JavierSaito, NorikoGarcia, AndresSaive, RebeccaGarcia, BelenSakaebe, HikariGarcia, Diana VilelaSakai, HiromiGarcia, IñakiSakai, JoeGarcía, NuriaSakar, MohanGarcía-Lastra, Juan MaríaSakthivel, TamilselvanGarcía-López, Elisa I.Sala, RobertoGarcía-Martinez, Joaquín C.Salahub, DennisGarcia-Pomar, Juan LuisSalaoru, IuliaGarcia-Sanchez, FranciscoSalas, CarlosGarcia-Segura, SergiSalguero, Tina T.Garcia-Torres, JoséSalinas Rodríguez, BeatrizGarcia-Verdugo, IgnacioSalleras, MarcGardiner, PhilipSalmas, ConstantinosGarg, AkhilSamad, Yarjan AbdulGargiulo, ValentinaSamanta, Pralok KumarGarmire, ElsaSamaras, IoannisGarro, NúriaSamoila, P. MugurelGarroni, SebastianoSamokhvalov, AlexanderGarshev, Alexey A.Samuel, TemesgenGartia, Manas RanjanSamulionis, VytautasGashti, Mazeyar ParvinzadehSan Fabian, EmilioGaskov, Alexander M.Sanchez, GoarGaspar, Maria ManuelaSánchez-García, DavidGatica, José M.Sánchez-Royo, Juan FranciscoGatti, AntoniettaSánchez-Tovar, RitaGatto, EmanuelaSancini, GiulioGauden, Piotr A.Sandanayaka, Atula S. D.Gaudry, ÉmilieSandre, OliverGautam, SiddharthSandri, GiuseppinaGavioli, LucaSandulescu, MadalinaGavrilov, AntonSangiovanni, DavideGebel, ThomasSankar, MeenakshisundaramGebicki, JacekSankaran, Kamatchi JothiramalingamGéczy, AttilaSannino, DianaGeier, Sebastian M.Sansone, AnnaGelbstein, YanivSansone, LuciaGeletii, YuriiSantamaria, RitaGellini, CristinaSantangelo, SaveriaGenovese, DamianoSanter, SvetlanaGentile, GennaroSanthanam, KSVGeorgantzinos, Stelios K.Santoni, AntoninoGeorge, ChandramohanSantoni, Marie-PierreGeorge, JanineSantos, AbelGeorge, Thomas F.Santos, ElizabethGeorgieva, RadostinaSantulli, CarloGeorgiou, T. K.Sanz-Garcia, AndresGeorgopanos, ProkopiosSaoud, KhaledGerasimov, Evgeniy Yu.Sapi, AndrasGerasimova, Yulia V.Sarantopoulou, EvangeliaGerislioglu, BurakSarasini, FabrizioGervais, FrançoisSarker, DipakGeso, MoshiSarker, SubrataGhanotakis, Demetrios FSarlin, EssiGheju, MariusSarma, RaktimGhibaudo, GérardSarris, IoannisGhica, DanielaSasai, YasushiGhica, Mihaela-VioletaSasaki, TomoyukiGhidelli, MatteoSashiwa, HitoshiGhittorelli, MatteoŠatínský, DaliborGhodake, GajananSato, KazunoriGhorbani-Asl, MahdiSato, KimiyasuGiacalone, FrancescoSatriano, CristinaGiacomazza, DanielaSaturnino, CarmelaGiamblanco, NicolettaSavelyeva, NataliaGiancane, GabrieleSawada, HideakiGiannakas, ArisSawada, ToshikiGiannazzo, FilippoSberveglieri, VeronicaGiannetti, AmbraScaffaro, RobertoGiapintzakis, John (Ioannis)Scaini, DenisGiaume, DomitilleScarano, DomenicaGibson, ChristopherScardaci, VittorioGiebeler, LarsScardi, PaoloGierszewska, MagdalenaScarpa, MarinaGigliotti, Casimiro LucaScarso, AlessandroGil, BernardScendo, MieczyslawGillan, Edward G.Schaefer, JenniferGiorcelli, MauroSchäfer, HelmutGiorgio, IvanScheicher, Ralph H.Girard, StevenSchepler, KennethGiraud, Marie NoëlleScherf, UllrichGirish, RughooburSchilirò, EmanuelaGirolami, MarcoSchintke, SilviaGîrțu, Mihai A.Schirhagl, RomanaGiubileo, FilippoSchlater, GuyGiulia, FioravantiSchmid, MarkusGiusti, IlariaSchmidbaur, HubertGizdavic-Nikolaidis, MarijaSchmidt, BernhardGlavina, DomagojSchmitt, KatrinGliemann, HartmutSchmutz, MarcGłuchowski, PawełSchnabel, ThomasGlukhova, Olga E.Schneider, RaphaelGlüsen, AndreasScholle, MarkusGmurek, MartaScholz, FerdinandGnade, Bruce E.Schueneman, GregGnaneswar Gude, VeeraSchulz, FlorianGnyba, MarcinSchulz, Sebastian AndreasGogneau, NoelleSchulze, MargitGogos, AlexanderSchweitzer, DietrichGolding, JonSciortino, AliceGoldoni, AndreaSciortino, LuisaGomes, Ana P.Scoponi, MarcoGomes, AndreiaScotognella, FrancescoGomes, JoãoScott, Amy M.Gomez de Salazar, José MaríaScrimin, PaoloGómez-Cámer, Juan LuisSecenji, AleksandarGómez-Graña, SergioSecu, Elisabeta-CorinaGomez-Ruiz, SantiagoSecu, M.Gonçalves, M. Sameiro T.Sedlačík, MichalGonçalves, RenatoSegawa, HiroyoGonzalez Vila, AlvaroSeifi-Mofarah, SajjadGonzález, IsraelSeisenbaeva, Gulaim A.Gonzalez, JoaquinSeitz, W. RudolfGonzalez, ZoraidaSekatski, SergueiGonzález-Cortés, AraceliSEKINE, TomohitoGonzález-Marcos, María P.Sel, OzlemGonzalez-Pedro, VictoriaSelimefendigil, FatihGonzalez-Velo, YagoSelke, MatthiasGoodwin, David G.Semenov, KonstantinGopalakrishnan, KothandamSemisalova, AnnaGopalan, SaianandSengupta, BidishaGoraus, JerzySeo, Hyung KeeGoreham, ReneeSequeira, César A.C.Gorgieva, SelestinaSerchi, TommasoGorji, Nima E.Sergey, MakarovGornakov, VladimirSerpooshan, VahidGorte, Raymond J.Serrà, AlbertGoryacheva, IrinaSerrano-Aroca, ÁngelGorzelanny, ChristianSessolo, MicheleGosselet, FabienSeynhaeve, Ann L. B.Goswami, SubhadipShabatina, TatyanaGoto, YosukeShacham-Diamand, YosiGouget, GuillaumeShadike, ZulipiyaGoula, Maria A.Shafiei, MahnazGouveia, Ricardo M.Shah, Furqan A.Govan, JosephShahjamali, MohammadGovind Rao, Vishal GovindShallcross, SamGowans, EricSham, T.K.Gracin, DavorShams, S. FatemehGraczyk-Jarzynka, AgnieszkaShang, LuoranGrama, SilviaShankar, EswarGrammatikopoulos, PanagiotisShapiro, BruceGrancha, ThaisShapovalov, VictorGranda, MarcosShapter, JoeGrande Tovar, Carlos DavidSharifi, TivaGranitzer, PetraSharkeev, YuriiGranozzi, GaetanoSharma, AnirudhGrassi, AlfonsoSharma, Piyush SindhuGrau, EtienneSharma, SaumyaGravalidis, ChristoforosSharma, SwatiGravel, EdmondShaw- Stewart, JamesGraves, JosephShayduk, RomanGrebel, HaimShaygan, MehrdadGrecco, GiuseppeShcherbakov, Maxim R.Gregoratti, LucaShchyglo, OlegGregori, AlbertoShearer, Cameron J.Grenha, AnaShehata, NaderGries, ThomasShehu, RafaelGrigoras, KestutisSheka, Elena F.Grigsby, Christopher L.Shekargoftar, MasoudGrijalvo, SantiagoShellaiah, MuthaiahGrishin, MaximShenashen, Mohamed A.Grossi, MarcoShenderovich, Ilya G.Grugeon, SylvieSher, Chin-WeiGrumezescu, AlexandruSheremet, MikhailGrunwald, RuedigerSheri, MadhuGruszecka, AgnieszkaSherrell, PeterGspann, ThuridShestakov, MikhailGuaraldo, Thais TassoShi, ZengqianGuarnieri, SimoneShields IV, C. WyattGuarrotxena Arlunduaga, Miren NekaneShilova, OlgaGucci, FrancescoShim, JunhoGuénin, ErwannShimada, ToshihiroGueorguiev, G.K.Shimanouchi, ToshinoriGueorguiev, GueorguiShimizu, FlavioGuerrero-Perez, M. OlgaShimizu, KazuoGuest, Jeffrey R.Shimobayashi, Shunsuke F.Guha, SuchiShimoi, NorihiroGuidotti, MatteoShimojo, MasayukiGuiot, CaterinaShimomura, OsamuGuisbiers, GregoryShimomura, TakeshiGulino, AntoninoShin, Bo-SungGumfekar, SarangShin, Su RyonGuo, Zhan-HuShinde, SudhirkumarGupta, ChiragShirokov, Vladimir B.Gupta, Ram K.Shokouhimehr, MohammadrezaGupta, SanjuShpacovitch, VictoriaGurikov, PavelShrestha, LokGuselnikova, Olga AndreevnaShtepliuk, IvanGusev, AlexanderSibillano, TeresaGusiatin, MariuszSiciliani, MarioGutekunst, Will R.Sideratou, ZiliGutiérrez, YaelSidorenko, AlexanderGutleb, ArnoSiegel, JakubGuzmán, EduardoŠiffalović, PeterGuzman, MarceloSigle, WilfriedGyörgy, EniköSignore, GiovanniH. Müser, Prof. Dr. MartinSikora, JanHa, MinjeongSikora, PawełHaber, LouisSilaghi-Dumitrescu, RaduHackenberg, StephanSilikas, NikolaosHaddadi, AbbasSilva, FilomenaHadipour, AfshinSilva, Maria JoaoHadjiargyrou, MichaelSilva, Teresa Moura EHadjikakou, Sotiris KSilvain, Jean-FrançoisHagita, KatsumiSilvan, Miguel MansoHahn, Konstanze R.Silveira, CéliaHähner, GeorgSilver, JackHak, SjoerdSilvestre, SamuelHalder, ArnabSim, UkHallett-Tapley, GenieceSima, FelixHama, SusumuSimeonidis, KonstantinosHämäläinen, JaniSimha Martynkova, GrazynaHampp, Norbert A.Simion, Cristian E.Hampton, JenniferSimmonds, Paul J.Hampton, MichaelSimões, SóniaHan, Dong-PyoSimon, SebastienHan, Dong-WookSimonescu, Claudia MariaHan, EricSimpson, David A.Hanaor, DorianSimserides, ConstantinosHanaor, Dorian A. H.Singh, Ashok K.Handoko, AlbertusSingh, DavidHanif, MuhammadSingh, GautamHaniu, HisaoSingh, GurwinderHao, JinsongSingh, JaiHara, KenjiSingh, SeemaHara, MasahiroSingha, SubhankarHaraguchi, NaokiSinha Ray, SumitHaralampos N, MirasSinha, JasmineHarja, MariaSinha, SudarsonHärmä, HarriSinha-Ray, SumanHarmandaris, Vagelis A.Siniscalco, DarioHarnois, MaximeSinn, GerhardHaro, MartaSiracusa, ValentinaHarrell, William R.Sirkar, KamaleshHarris, PeterSirutkaitis, ValdasHartnagel, HansSisti, LauraHasanzadeh Kafshgari, MortezaSitarz, MaciejHashimoto, MakotoSiwińska-Ciesielczyk, KatarzynaHatachi, YukimasaSkorik, Yury A.Hatada, RurikoSkowroński, ŁukaszHatakeyama, KazutoSkwarek, EwaHatta, AkimitsuŚlebarski, AndrzejHattar, KhalidSloan, JeremyHattori, Azusa N.Slobodian, PetrHattori, Haroldo T.Sloma, MarcinHatziantoniou, SophiaSłomkowski, StanislawHatzikraniotis, EvripidisSmani, YounesHawash, ZaferSmått, Jan-HenrikHawes, Chris S.Smith Callahan, Laura A.Hayakawa, TohruSmith, AdamHayashi, K.Smith, BryanHayashi, TakuyaSmith, Henry I.He, AinaSmith, Robert E.He, DelongSmolyanitsky, AlexHe, JieSnee, PrestonHedberg, JonasŠob, MojmírHefferon, Kathleen LauraSobolev, KonstantinHegedűs, CsabaSobotta, LukaszHegedűs, LászlóSocol, MarcelaHejna, AleksanderSogorb Sánchez, Miguel ÁngelHellweg, ThomasSohn, YoungkuHelsch, GundulaSola, RamonaHémadi, MiryanaSoldano, CaterinaHempel, MartinSolomonov, Alexey V.Hemraj-Benny, TirandaiSon, DongheeHenderson, Luke C.Son, MoonHendrix, Justin W.Son, Seung UkHerckes, PierreSonde, SushantHermerschmidt, FelixSøndergaard, ThomasHermoso-Orzáez, Manuel JesúsSong, SunghoHerranz, FernandoSong, Young MinHerrasti Gonzales, PilarSoon, Jong JeongHerrera-Melián, José AlbertoSorrentino, AndreaHerrmann, AndreasSotgiu, GiovanniHeuken, MichaelSotiriou-Leventis, CharikliaHibino, TakashiSotiropoulos, SotirisHigashi, MasanobuSoto, PaulHigginbotham Duque, Amanda L.Sotome, MasatoHill, DanielSowa, IreneuszHilliou, LoicSpagnuolo, GianricoHinchet, RonanSpanopoulos, IoannisHinderliter, BrianSpeghini, AdolfoHintzen, HubertusSperanzini, EmanuelaHippler, RainerSpieß, LotharHirano, AtsushiSpirk, StefanHirsch, ThomasSportelli, Maria ChiaraHix, GarySprio, SimoneHjelme, Dag RoarSprynskyy, MyroslavHnát, JaromírSrinivas, KeerthiHo, DanielSrivastava, G.P.Ho, TuananhSt John, James A.Hobbie, Erik K.Stadnichenko, AndreyHodoroaba, Vasile-DanStafeev, SergeyHoffman, JasonStafford, LucHoffmann-Eifert, SusanneStahl, FrankHofman-Caris, RobertaStamate, EugenHogenEsch, HarmStamatis, HaralambosHOLADE, YaoviStan, MirunaHoller, StephenStanca, Sarmiza ElenaHolmes, MarkStańczyk, Wlodzimierz A.Holynska, MalgorzataStaneva, DesislavaHomaeigohar, ShahinStaruch, MargoHonda, TakashiStassen, IvoHong, Suck WonStaszak, KatarzynaHong, SukjoonStavenga, Doekele G.Hoppstädter, JessicaStedman, KennethHorak, DanielStefan, NeatuHorino, YoshikazuStefanelli, ManuelaHornak, JaroslavŠtefanić, Petra PeharecHorovistiz, AnaStegmann, PhilippHorszczaruk, ElżbietaSteinbach-Rankins, JillHoskins, ClareSteinfeldt, NorbertHospodková, AliceSteinhauer, StephanHossain, Shahriar A.Stellato, FrancescoHou, HarveyStelmachowski, PawełHou, Shuhn-ShyurngStepanov, Gennady V.Housecroft, CatherineStephan, HolgerHouska, JiriStępniak, IzabelaHowes, PhilipStępniowski, WojciechHoyos, PilarSteup, MartinHristoforou, EvangelosStewart, George C.Hsiang, Hsing-IStievano, LorenzoHsiao, Chung-DerStine, Keith J.Hsiao, Vincent K.S.Stinespring, CharterHsieh, Chien-KuoStiufiuc, RaresHsieh, Chien-WenStobiecka, MagdalenaHsu, Yu-KueiStöckelhuber, Klaus WernerHsueh, Yi-HuangStoikov, Ivan IvanovichHu, Teh-MinStojek, ZbigniewHuang, Chia-HuaStolarczyk, AgnieszkaHuang, Chih-ChiaStoleru, ElenaHuang, Chih-ChingStolojan, VladHuang, Kuo-ChengStrankowski, MichalHuang, RuomengStrelniker, YakovHuang, Shih-YungStride, John ArronHuang, Wen ChangStrini, AlbertoHubbe, Martin A.Strunk, JenniferHueso, Jose L.Strzelecka-Kiliszek, AgnieszkaHughes, Robert A.Stylianakis, Minas M.Huguet, Silvia SoriaSu, Chia-HaoHumar, MihaSu, Wen-TaHumbert, ChristopheSuárez, IsaacHUMELNICU, DoinaSuarez, SebastianHung, Tai-FengSuber, LorenzaHuple, DeepakSuckeveriene, RanHur, JaehyunSugai, YuichiHúska, DaliborSugawa, KosukeHussain, ManwarSugiura, YukiHuttel, YvesSuh, JoonkiHuynh, Thanh-CanhSukhov, SergeyHwang, Chi-SunSumboja, AfriyantiHwang, SungyeonSun, An-ChengHwang, Taek YongSun, ConroyHytönen, VesaSun, HaoIacovella, Christopher R.Sun, HongyuIanchis, RalucaSun, WujinIannazzo, DanielaSun, XiangchengIatrou, HermisSundqvist, JonasIatsunskyi, Igor RSunna, AnwarIbrahim, ToniSurmenev, Roman A.Ichikawa, MasakazuSuryanto, BryanIchikawa, TakahiroSuzuki, ShinyaIchikawa, YoSuzuki, YoshioIemmo, LauraSuzuki, YukiIervolino, GiuseppinaSvarnas, PanagiotisIgnaszak, AnnaSvensson, Ann MariIgnat, MariaSvorc, LubomirIhlemann, JürgenSwaaij, René VanIjima, HiroyukiSweetman, MartinIjiro, KuniharuSykora, MilanIlahi, BouraouiSyrovy, TomasIlanchezhiyan, PugazhendiSzabó, TamásIlg, PatrickSzałowski, KarolIllés, BalázsSzczerba, JacekIlles, ErzsebetSzczurek, AndrzejIllés, ErzsébetSzekely, GyorgyImamura, GakuSzepvolgyi, JanosInada, RyojiSzewczyk, RomanInagaki, NorihiroSzilágyi, Imre MiklósInfantes-Molina, AntoniaSzilvási, TiborInoue, ShuheiSzőri, MilánIoannides, TheophilosSzperlich, PiotrIoannis, KonidakisSzukalski, AdamIonete, Eusebiu IlarianSzybowicz, MiroslawIonita, PetreSzymańska, RenataIram, SurtajSzymborski, TomaszIrena, KratochvílováSzymczyk, AnnaIrene Urbieta Quiroga, AnaTabarraei, AlirezaIrrera, AlessiaTaboryski, Rafael J.Irving, TomTadanaga, KiyoharuIsaacs, MarkTagliatesta, PietroIsaad, JalalTaglietti, Angelo MariaIsakov, DmitryTahergorabi, RezaIsalgue, AntonioTaheri, ShimaIseppi, RamonaTai, Cheuk-WaiIshihara, KazuhikoTailhades, PhilippeIshii, AyumiTakafuji, MakotoIshikawa, KenjiTakagaki, AtsushiIshikawa, RyoTakahashi, KazuyukiIshikawa, TadahikoTakahashi, KeisukeIshikawa, YasuakiTakahashi, YasuoIshizaki, KenjiTakahiro, KatsumiIslamoglu, TimurTakamatsu, SeiichiIsland, J. O.Takano, ToshiyukiItina, Tatiana E.Takashima, ToshihiroIto, ShosukeTakayoshi, SuzukiIto, TakeruTakeoka, ShinjiIto, TakeshiTakeshita, FumitakaIto, YoshihiroTakeshita, SatoruItoh, TamitakeTakeuchi, TomonariItsuno, ShinichiTalaat, AhmedIvanets, AndreyTalapaneni, SiddulunaiduIvanov, Ilia N.Taleb, AbdelhafedIvanova, SvetlanaTallarico, MarcoIwan, AgnieszkaTampucci, SilviaIwanaga, MasanobuTamulevičienė, AstaIwata, TatsuyaTanabe, KatsuakiIzadyar, AnahitaTanaka, ShinjiJ. Jankiewicz, BartłomiejTanaka, TomonariJabłońska, BeataTangoulis, VassilisJacak, JarosławTanikawa, TomoyukiJacek, ŻmojdaTański, TomaszJadczak, Joanna N.Tao, ShanwenJadwicienczak, WojciechTappertzhofen, StefanJain, AnkurTaratula, OlenaJakieła, SławomirTard, CédricJakšić, OlgaTardy, BlaiseJakubik, Wiesław P.Tasca, FedericoJakubowicz, JarosławTasis, DimitriosJakubowski, WitoldTateishi-Karimata, HisaeJamróz, PiotrTatullo, MarcoJamting, AsaTavakoli, JavadJanas, DawidTavares, AnthonyJanczarek, MarcinTayabali, AzamJänes, AlarTayade, RajeshJang, ChangheuiTayeb, Ali H.Jang, Jeong GookTaylor, HaydenJang, Tae-SikTcherdyntsev, Victor V.Jankauskaite, VirginijaTchoukov, PlamenJanovák, LászlóTeghil, RobertoJanovec, JozefTehranchi, AmirhosseinJanowska, IzabelaTeixeira, JorgeJarosz-Wilkołazka, AnnaTejedor, José Antonio GómezJaszcz, KatarzynaTelegdi, JuditJavier, Sánchez-NievesTelukutla, Srinivasa ReddyJeeyoung, YooTeodorescu, FlorinaJeffryes, ClaytonTeodorescu, Valentin ŞerbanJena, HimanshuTerao, KenJeon, Ju-WonTereshina-Chitrova, E.A.Jeon, SeokwooTERMENTZIDIS, KonstantinosJeong, Hyung MoTeymourian, HazhirJeong, Jong-RyulThai Duy, LeJeong, Nak CheonThakur, GarimaJeong, Sang MoonThakur, SachinJeong, SanghwaThakur, Vijay KumarJewell, Eifion HuwThang, Tran QuyetJha, BirendraTheres Baby, TessyJha, Kshitij C.Thijssen, JosJha, Ramesh K.Thomas, Iorwerth O.Jiang, ChangyunThomas, T.J.Jiang, XuchuanThrivikraman, GreeshmaJicsinszky, LászlóThygesen, Kristian SommerJimenez Ballesta, Ana EvaTibbetts, Katharine MooreJiménez, JuanTiberto, PaolaJiménez-Saelices, ClaraTiemann, MichaelJimenez-Villacorta, FelixTijing, LeonardJin, Hyeong MinTikhonravov, AlexanderJing, QingshenTiller, Joerg C.Jisha, Chandroth PannianTimlin, Jerilyn A.Johanson, UrmasTimperman, LaureJohansson, ChristerTiseanu, CarmenJohnson, DavidTitvinidze, IrakliJohnston, Linda J.Tkach, AlexanderJolly, PawanTkachenko, NikolaiJones, LatheTobaldi, David MariaJönsson-Niedziolka, MartinToci, GuidoJoo, Ji BongTofan, LaviniaJordan, AlexToffanin, StefanoJørgensen, BodilToietta, GabrieleJoshi, RakeshTojo, ConchaJoshi, RavindraTokumoto, YukiJoshi, SanketTolochko, OlegJoshi, TanmayaTomalia, DonaldJotshi, ChandTombelli, SaraJoubert, OlivierTomer, Vijay K.Jouini, NoureddineTomita, SatoshiJu, HeongkyuTomita, ShunsukeJulien, Christian M.Tomm, JensJun, Bong-HyunTomov, Rumen I.Jun, KimTomovska, RadmilaJun, Young-SiTomšič, BrigitaJung, JaehanToncelli, ClaudioJung, JaehanToncheva, AntoniyaJung, Jong HwaTondi, GianlucaJung, Yeon-GilTonelli, DomenicaJung, Yong ChaeTonti, DinoJuodkazis, SauliusTopoglidis, EmmanuelJurado Sánchez, BeatrizTopuz, FuatJurado, Amalia S.Torgersen, Maria LyngaasJurca, TitelTorigoe, KanjiroJursich, GregoryTorii, ShuichiKabanos, ThemistoklisTorimoto, TsukasaKachynski, AliaksandrTornabene, FrancescoKačiulis, SauliusTorre, Maria LuisaKaczmarek, Anna M.Torreggiani, ArmidaKaczorek, EwaTorres, DanielKadam, AvinashTorres-Lagares, DanielKadkhodazadeh, ShimaTorsten, GranzowKado, YuyaTortora, LucaKahlert, HeikeTotaro, GraziaKajimoto, ShinjiTotaro, MassimoKajitani, TakashiToudert, JohannKako, TetsuyaTownley, Helen E.Kalantar-zadeh, KouroshToyao, TakashiKalanur, Shankara SharanappaTraini, ToninoKalas, WojciechTrammell, Scott A.Kalbáčová, Marie HubálekTran, FabienKaliginedi, VeerabhadraraoTretiakov, KonstantinKalisch, HolgerTribello, GarethKallitsis, Joannis K.Trindle, CarlKalogirou, OrestisTrinh, ThuatKaluža, LuděkTripathi, Jitendra KumarKamada, KaiTripathi, Kumud MalikaKamal, Musa R.Trivedi, RahulKamali, SaeedTrivellin, NicolaKamanina, Natalia V.Trocha, PiotrKameyama, AtsushiTrubiani, OrianaKamiguchi, SatoshiTrukhanov, AlexeiKamimura, TakafumiTrukhanov, SergeiKamińska, AgnieszkaTruong, Nghia P.Kamitaka, YujiTruong, Vi-KhanhKampouris, DimitriosTrzeciak, AnnaKanaev, AndreiTsai, Chen-YenKanazawa, KenTsai, Pei-WeiKanda, KazuhiroTsamis, ChristosKaneco, SatoshiTsay, Chien-YieKaneti, Yusuf ValentinoTseng, Hui-hsinKang, YoungsooTseng, Zong-LiangKanik, MehmetTsitsilianis, ConstantinosKantartzis, Nikolaos V.Tso, Chi YanKapcia, Konrad JerzyTsoi, James Kit-honKaplan, AndreyTsou, Nien-TiKaragiannakis, GeorgeTsow, Francis (Tsing)Karakashev, StoyanTsujimoto, TakashiKarakatsanis, Nikolaos A.TSUTSUI, MakusuKarapanagiotis, IoannisTsuyumoto, IsaoKareiva, AivarasTsuzuki, TakuyaKargl, RupertTsvetkov, NikolaiKarle, TimothyTsyganenko, Alexey A.Karnahl, MichaelTubaro, CristinaKároly, LázárTudorache, FlorinKarousou, EugeniaTudoroiu, NicolaeKarpov, VictorTulliani, Jean MarcKarpukhina, NataliaTumarkin, AndreyKartashov, Yaroslav VTumuluri, UmaKarwowska, EwaTung, Shih-HuangKashiwagi, ShinichiroTung, Tran ThanhKasnatscheew, JohannesTurco, AntonioKasprowicz, DobrosławaTurco, RosaKatal, RezaTurkowski, Volodymyr M.Kataoka, YusukeTurner, NeillKatin, Konstantin P.Tuszynski, Jack A.Kato, HaruhisaTuti, SimonettaKatsura, ShinjiTuttle, BlairKatunin, AndrzejTyliszczak, BożenaKaunas, RolandTylkowski, BartoszKauppinen, Esko I.Tzetzis, DimitriosKaur, AmandeepTzschucke, ChristophKaushik, NagendraUccelletti, DanielaKawabe, YutakaUchida, SatoshiKawakami, HiroshiUchida, TetsuyaKawakita, HidetakaUddin, AshrafKehl, FlorianUeda, TaroKelder, Erik M.Ujihara, MasakiKeller, AdrianUkrainczyk, NevenKelley, DavidUl Haq, RizwanKelley, StevenUlewicz, MalgorzataKendrick, EmmaUm, Han-DonKerdjoudj, HalimaUmek, PolonaKetov, SergeyUmerska, AnitaKhadka, Dhruba B.Umrath, StefanKhalid, AtaUnnithan, Ranjith RajasekharanKhan, Omar F.Unno, MasafumiKhan, ZiauddingUr, Soon-ChulKhanal, RabiUrban, OtmarKharel, ParashuUrbanczyk-Lipkowska, ZofiaKhashayar, PatriciaUrbani, MaxenceKhisamutdinov, EmilUrbassek, HerbertKhodadoust, Amid P.Urbieta, AnaKhodaparast, GitiUsov, NikolaiKhokhlova, Marina D.Usui, HidetomoKhoo, Bee LuanUznanski, PawelKhorasani, SinaVacca, AnnalisaKhulbe, Kailash ChandraVaculovičová, MarketaKhyzhun, O.Yu.Vadahanambi, SridharKiciński, WojciechVadas, Timothy M.Kiefer, RudolfVahabi, HenriKim, Bo-HyunVaiano, VincenzoKim, BojeongVaidyanathan, SriramKim, BongjuVakros, JohnKim, Chang-HyunValais, IoannisKim, Dai-SikValentini, FedericaKim, Do-GunVallée, ChristopheKim, DonghyunValov, IliaKim, DonginVan Dyke, Michael W.Kim, Dong-Joovan Ommen, J. RuudKim, Dong-jooVan Schepdael, AnnKim, DongwookVan Straalen, Nico M.Kim, FelixVancaeyzeele, CédricKim, Gue-HyunVanetsev, AlexanderKim, HangunVankayala, RavirajKim, Hyun JaeVaradwaj, Pradeep R.Kim, Hyun TakVarikuti, SanjayKim, HyungwooVarma, Rajender S.Kim, HyunsungVarsano, DanieleKim, Ii TaeVashist, ArtiKim, JaeyounVasilache, DanKim, Jeong-ChulVasilakaki, MariannaKim, JeonghunVasile, Bogdan StefanKim, JeonghyunVasile, CorneliaKim, JeongkwonVasilyev, AlexanderKim, Jong HyunVasquez, YolandaKim, Jung KyuVassilis J, InglezakisKim, Jun-HyunVattikuti, Surya Veerendra PrabhakarKim, Keun SuVaz, CarlosKim, Keun-SooVaz, Irene PinaKim, Ki BuemVaz-Moreira, IvoneKim, Ki KangVecino, XanelKim, Kyong HonVedarajan, RamanKim, Moon DeockVedraine, SylvainKim, Moon IlVédrine, JacquesKim, MyungwoongVejpravova, JanaKim, Nam KyeunVekinis, GeorgeKim, Ok-SuVelasco-Vélez, Juan JesúsKim, Prof. Dr. ChoongikVelazquez-Perez, Jesus EKim, Sang-JaeVeleeparambil, ManojKim, SeokminVelíšek, JosefKim, Seong-GonVelonia, KellyKim, SeongsinVenditti, IoleKim, SeunghyunVeremchuk, IgorKim, Soo YoungVereshchaka, Alexey A.Kim, Tae GeunVerestiuc, LilianaKim, TaehoonVerma, AmitKim, Tae-HyungVerma, SannyKim, Yoong AhmVermorel, RomainKim, YounghoonVernardou, DimitraKIM, YUNAVeronesi, GiuliaKimata, MasafumiVeronis, GeorgiosKioseoglou, GeorgeVertruyen, BenedicteKirchner, RobertVescan, AndreiKirihara, SoshuVetter, Stefan W.Kirm, MarcoVeziroglu, SalihKirste, RonnyVicenzo, AntonelloKirszensztejn, PiotrVictor J., RicoKiryukhantsev-Korneev, Ph.V.Videira, Romeu A.Kiss, JánosVidic, JasminaKitagawa, JiroVieru, DumitruKitahama, YasutakaVijayaraghavan, VenkateshKitajou, AyukoVikraman, DhanasekaranKitamura, TakayukiVilà, AnnaKitazawa, TakafumiVilanova, XavierKityk, IwanVilardi, GiorgioKlajnert-Maculewicz, BarbaraVillaverde, Juan JoséKlebert, SzilviaViña Olay, JaimeKlemm, RichardVinardell, MariaKlinkova, AnnaVinci, GiulianaKliros, George SVinnik, DenisKłonkowski, Andrzej M.Vinogradov, VladimirKluczka, JoannaVinokurov, VladimirKnibbe, RuthVirginie, PONSINETKo, ChanghyunViskadourakis, Zacharias A.Ko, Dong-KyunViter, RomanKo, SeunghwanVitiello, GiuseppeKobayashi, AtsushiVitrik, Oleg BorisovichKobayashi, JunVittori Antisari, MarcoKobayashi, RikaVittorio, OrazioKobayashi, ShunsukeVivas Mejia, PabloKoblischka, Michael R.Viviani, MassimoKobtsev, SergeyVivo, PaolaKoch, ReinholdVlachos, NicholasKochanowicz, MarcinVlachou, MarilenaKoci, KamilaVladyslav, VakarinKoczorowski, TomaszVlaskin, MikhailKoduru, JanardhanVohra, VarunKoenders, E.A.B.Voicu, Stefan IoanKoetz, JoachimVoliani, ValerioKoh, AhyeonVolk, DavidKöhler, KarstenVolker, KörstgensKöhler, RobertVolkov, YuriKohler, SigmundVolochaev, V. A.Kohtani, ShigeruVoronov, AndriyKOINKAR, PANKAJVorontsov, AlexanderKojima, ToshikatsuVourdas, NikolaosKokado, KentaVoutetaki, Maria–StylianiKoksharova, Olga A.Voyiatzis, GeorgeKolanjiyil, Arun VargheseVozzi, GiovanniKolaric, BrankoVranes, MilanKolasinski, Kurt W.Vrcek, Ivana VinkovicKolosnjaj-Tabi, JelenaVriesekoop, FrankKolpashchikov, DmitryWachowski, SebastianKoltai, JánosWachtveitl, JosefKonarova, MuxinaWada, TohruKondo, MioWaite, Matthew M.Kong, JaeminWakiya, NaokiKonshina, ElenaWal, R. L. VanderKonsolakis, MichalisWalach, WojciechKonstantinov, Spiro M.Walde, PeterKontonasaki, EleanaWalia, SumeetKontziampasis, DimitriosWallace, David R.Koo, Hee-beomWallace, Kenneth N.Kooij, E. StefanWang, Chih-FengKooyman, PatriciaWang, Chiu-YenKopecký, DušanWang, Di-YanKopel, PavelWang, Dong HwanKoplitz, BrentWang, JiangxinKoponen, AnttiWang, ShapengKordas, KrisztianWang, SongcanKordesch, Martin E.Wang, TaoKoren, KlausWang, XiaoleiKőrösi, LászlóWang, XiaoweiKorzeniewska, EwaWang, YuKoshimizu, MasanoriWang, Yun-CheKoshizaki, NaotoWark, Alastair W.Kosinova, Marina L.Wark, MichaelKosinski, PawelWarwick, Michael E. A.Kostiainen, MauriWąsik, Tomasz J.Kostin, Gennadiy A.Wasilewski, TomaszKoszegi, TamasWatanabe, FumiyaKotek, JanWatanabe, MasahitoKoter, StanislawWatanabe, MasatoshiKotsiantis, SotirisWatkins, A. NealKotsuchibashi, YoheiWatras, AdamKottapalli, Ajay Giri PrakashWatzky, MurielleKou, TianyiWautier, Jean-LucKoubiti, MohammedWdowin, MagdalenaKoumoulos, Elias P.Webber, GrantKounatidis, IliasWeber, MatthieuKourkoumelis, NikolaosWęglarz, WładyslawKoutavarapu, RavindranadhWei, GangKoutselas, IoannisWeichert, JameyKoutsogeorgis, DemosthenesWeigand, R.Koutzarova, TatyanaWeiss, RichardKouzu, MasatoWeller, Michael G.Kovács, ZoltánWen, Amy M.Kovács, ZsoltWhitby, Catherine PKovalcik, AdrianaWhite, MarkKovalevsky, Andrei V.Whitehead, Debra E.Kowalak, StanisławWidenmeyer, MarcKowalczuk, DorotaWie, Myung-BokKowalska, EwaWieczorek, KingaKozak, MartinWieczorek, Władysław G.Koziol, MateuszWiemann, MartinKozliak, Evguenii I.Wieszczycka, KarolinaKramer, PhillipWiglusz, Rafał J.Krasnoslobodtsev, Alexey V.Wilhelm, ReneKratzer, MarkusWilkins, Richard TKrause, Hans-JoachimWilliam Coleman, AnthonyKrawiec, MariuszWilliams, GarethKreouzis, TheoWilson, Andrew J.Kriegner, DominikWilts, BodoKrim, JacquelineWinkler, KKrisyuk, VladislavWiśniewski, MarekKrólicka, AgnieszkaWittemann, AlexanderKrólicka, AleksandraWitzel, BerndKroll, PeterWłodarczyk, PatrykKrstulović, NikšaWojcieszak, RobertKrysinski, PawelWojcik, JanuszKrzanowski, JamesWojnarowicz, JacekKrztoń-Maziopa, AnnaWong, Ka-LeungKubo, MasatakaWong, PatrickKubo, TakanoriWong, Roong JienKubota, YoshikiWon-Yeop, RhoKucherik, AlexeyWoo, Yun SungKuchler-Bopp, SabineWoodward, Robert T.Kuchmizhak, AleksandrWorrall, StephenKuckling, DirkWorsley, Marcus A.Kudaibergenov, SarkytWróbel, JanKuddannaya, ShreyasWu, Chia-WenKudelski, AndrzejWu, DonghaiKudłak, BłażejWu, Mao-SungKudlek, EdytaWu, Yu-TseKudryashov, Sergey I.Wujcik, Evan K.Kujawa, JoannaWuttke, StefanKujawski, WojciechWycisk, RyszardKukli, KaupoWyman, IanKukovec, Boris-MarkoWysokowski, MarcinKukułka, WojciechXia, LanKulinich, SergeiXie, HaiboKulinsky, LawrenceXifré-Pérez, ElisabetKulka, MichalXu, TingtingKumagai, SeijiXu, XiaoxueKumar, AnilYadav, Hemraj MKumar, NarendraYadav, Raghvendra SinghKumar, NareshYadav, SushmaKumar, PawanYadavalli, Nataraja SekharKumar, PrasunYadgarov, LenaKumar, VipinYagoubi, SaïdKumar, VivekYahiaoui, RiadKumeria, TusharYakimov, E.B.Kumi-Diaka, JamesYakimova, RositsaKunsagi-Mate, SandorYalcinkaya, FatmaKuo, Chi-ChingYamada, ToshishigeKuo, Dong-HauYamaguchi, AritomoKuo, ShiaoweiYamaguchi, MakotoKuo, Tsung-RongYamaguchi, SeijiKupka, TeobaldYamamoto, GoKuřitka, IvoYamamoto, YusukeKurlyandskaya, Galina V.Yamato, TakehikoKuroda, TakashiYamauchi, YusukeKurzatkowska, KatarzynaYáñez-Sedeño, PalomaKusior, AnnaYáñez-Vilar, SusanaKusko, MihaelaYang, C.-F.Kuśmierek, KrzysztofYang, En-Che YangKustov, LeonidYang, HsiharngKustrowski, PiotrYang, Jen-ChangKuttner, ChristianYang, LilyKuzhir, PavelYano, MitsuakiKuzhir, Polina P.Yao, QiaofengKvashnin, Dmitry G.Yasui, KyuichiKvitek, LiborYe, LongKwang, HeoYee, Ki-JuKwon, Hyuck-InYehezkeli, OmerKwon, Ki-YoungYentekakis, IoannisKwon, Nam HeeYeo, Boon SiangKwon, Oh JoongYeom, JunghoonKwon, Oh SeokYeom, JunseokKwon, Soon-HongYerushalmi-Rosen, RachelKwon, Soon-HongYeum, JeongKwon, Tae-YubYi, Gi-RaKyzioł, AgnieszkaYildirim, AdemLa Deda, MassimoYilmaz, MehmetLa Flor, Silvia DeYokota, ShingoLa Parola, ValeriaYoo, Dong JinLa Spada, LuigiYoo, DongwonLaarmann, TimYoon, Dae SungLabuta, JanYoon, HyeonseokLacabanne, ColetteYoon, MinyoungLacarbonara, WalterYoon, SorahLachman-Senesh, NoaYoshimatsu, KoheiLacivita, ValentinaYoshimoto, MakotoLacrampe, Marie-FranceYoshino, ToshihikoLadero, MiguelYou, JungmokLaforge, François O.You, ShujieLahiri, AbhishekYounis, AdnanLahtinen, JoukoYperman, JanLai, Chih-HsienYu, HakkiLai, Jui-YangYu, Jae WoongLai, Ngoc DiepYu, JiashingLai, Wing-FuYu, LuLai, Yi ShengYu, TaekyungLaidani, Nadhira BensaadaYu, Xiao-YingLaïk, BarbaraYuan, KuoLaity, Peter RYuba, EijiLakshminarayana, PolavarapuYudasaka, MasakoLalatonne, YoannYuguchi, YoshiakiLambrecht, WalterYuldashev, Sh.Lampakis, DimitriosYum, Jun HoLamperska, WeronikaYun, KyusikLamprou, Dimitrios A.Yun, Seok JoonLan, DongchenYun, Young SooLanc, DomagojYurukcu, MesutLanceros-mendez, SenentxuYusenko, Kirill V.Lange, HolgerZabihi, OmidLanger, Jerzy JZaccheroni, NelsiLanzi, MassimilianoZacharatos, FilimonLaptev, AlexanderZaitsu, Shin-ichiLarese, FrancescaZakharenko, A.M.Larraneta, EnekoZamani, AkramLartigue, LenaicZamaratskaia, GaliaLaskowski, ŁukaszZambon, DanielLaso, ManuelZandvliet, HaroldLatonen, Rose-MarieZannotti, MarcoLatosińska, Jolanta NataliaZanotti, Jean-MarcLatouche, CamilleZappa, DarioLatterini, LoredanaZaraska, LeszekLattuada, MarcoZarejousheghani, MashaalahLau, Woei JyeZarifi, Mohammad H.Laurencin, Cato T.Zarkadoula, EvaLaurenti, MarcoZarrelli, MauroLavoine, NathalieZavaleta, CristinaLayek, BuddhadevZawala, JanLazzara, GiuseppeZazpe, RaulLe Donne, AlessiaZekonyte, JurgitaLe Ouay, BenjaminŻelechowska, KamilaLeavesley, DavidZelzer, MischaLeBlanc, GabrielZemek, JosefLechanteur, AnnaZeng, HuangLechner, Bob-DanZeng, ShuwenLeclerc, CoreyZergioti, IonnaLecomte, PhilippeZhang, QiangzheLedwon, PrzemyslawZhang, R. Y.Lee, ChangwonZhang, RunLee, Che-HsinZhang, XiangLee, Chi HwanZhang, XinLee, Chia-HungZhang, XuefengLee, Chia-HungZhang, YueLee, Dong HyunZhao, DonggaoLee, DongkyuZhao, LianfengLee, DoojinZhao, YudaLee, EungjeZheng, XintingLee, EunjungZhi, ChunyiLee, EunsongyiZhitova, ElenaLee, GeunsikZhou, YiqunLee, HwasungZhu, CanLee, IlkeunZhu, JiadengLee, IlkeunZhu, JieLee, Jae SungZhuravlev, VictorLee, JaejongZi, YunlongLee, Jea UkZiegler-Borowska, MartaLee, JihoonZielinska, BeataLee, Ju-HyuckZielińska-Jurek, AnnaLee, Jung SeokZieliński, AndrzejLee, KangwonZiemkowska, WandaLee, Kee-SunZignani, Sabrina CampagnaLee, Kyung-JinZille, AndreaLee, MinbaekZiora, ZytaLee, Rong-HoZitka, OndrejLee, SejoonZografopoulos, Dimitrios C.Lee, Seok JaeZolfagharian, AliLee, Seok WooZubia, DavidLee, ShiwooZubrik, AntonLee, TaekZukowski, WitoldLee, Woo-KyungZuo, LijianLee, Young-ChulZuorro, AntonioLeem, Jung WooZur, LidiaLefebvre, JacquesŻyła, GawełLefferts, Leon

